# SARS-CoV-2 and reproductive system: a scientometric study

**DOI:** 10.3389/frph.2026.1844245

**Published:** 2026-05-14

**Authors:** Linghan Zhang, Minkai Wang, Huijuan Zhang, Genhong Mao

**Affiliations:** 1 Department of Reproductive Medicine, The Second Affiliated Hospital of Zhengzhou University, Zhengzhou, China; 2 Department of Neurosurgery, The First Affiliated Hospital of Zhengzhou University, Zhengzhou, China; 3 Department of Gastroenterology, The First Affiliated Hospital, Hainan Medical University (Hainan Academy of Medical Science), Haikou, China

**Keywords:** female fertility, genital cells, reproductive system, SARS-CoV-2, scientometric study, thematic trends

## Abstract

**Objectives:**

Growing evidence, such as Long COVID, suggests that the interaction and underlying mechanism between SARS-CoV-2 and human cells, especially the long-term effect on the reproductive system, remain poorly understood, which should raise high public health concern. This study aimed to enhance understanding of the scientific development status in this field of the viral infection and the human genital system, and to explore key hotspots, thematic trends, major issues through a scientometric analysis of the relevant literature.

**Methods:**

This study retrieved the literature (2020–2025) on association between SARS-CoV-2 and the genital system from three databases, Web of Science Core Collection (WOSCC), Scopus, and PubMed, and performed comprehensive scientometric analysis of the literature to explore the bibliometric distribution, thematic trends and the major problems in this area using Bibliometrix/BiblioShiny.

**Results:**

A total of 2,354 publications from 2020 to 2025 were included in the analysis. The annual production of the publications has shown a declining trend since 2022, and is predicted to reach nearly zero by 2030. The most relevant journal was Mathematical Biosciences and Engineering. Wang X. and Wang Y. were the most prolific authors. China and the USA had the highest production of documents, while Sweden and Portugal had the highest average article citations. The most global cited document was “Cytokine Storm” (Fajgenbaum D, 2020, New Engl J Med). The most frequent keywords were covid-19, human, sars-cov-2, cell proliferation, pandemic, and basic reproduction number. Etiology, antigen presentation and sperm viability have been the newly emerging trend topics since 2025. A thematic map shows that the cluster of the keywords closely related to the impact of SARS-CoV-2 on reproductive system was located in the lower left quadrant, indicating that the themes associated with these keywords appeared early but remain underdeveloped. The USA, China, Italy and Germany conducted the most research collaborations, while most African, Latin American, and Asian countries were rarely involved.

**Conclusion:**

This report provides comprehensive insights, including the latest macroscopic perspectives, references, and practical guidance, for shaping research strategies, managing public health resources, and fostering scientific collaborations, featuring novel viewpoints that merit attention in the field.

## 1 Introduction

The novel coronavirus SARS-CoV-2 has caused more than 700 million infections and 7 million deaths throughout the world (1). This viral infection can lead to COVID-19, affecting multiple organ systems, not only involving respiratory and cardiovascular systems (2), but probably also the reproductive system. Although increasing evidences, including detection of the receptors like ACE2 and viral mRNA in testis, and adversely affected sperm quality in COVID-19 cases (3, 4), have suggested the potential of SARS-CoV-2 to invade human germinal cells and impair reproductive function, the impact of this infection on human reproductive cells and pathogenesis are undefined. Accompanying reports on post-acute sequelae of COVID-19 or long COVID (LC) with persistent immune, neurological, and cardiovascular symptoms, the influence of SARS-CoV-2 infection on human fertility should attract public health concern (5, 6). Given the potential implications of this viral infection for human reproductive health, it is crucial to accurately assess the long-term consequence, determine the reversibility and precise recovery timeline of such damage, and evaluate post-infection reproductive health parameters (4–6). Therefore, further ascertaining effect of SARS-CoV-2 and underlying mechanism on genital cells is important for solving this global public health issue.

Scientometrics combines mathematical, bibliographical, and statistical methods for quantitative analyses of all knowledge carriers, which has advantages of evaluating academic achievements and predicting research trends (7). Bibliometrix is an R-software package designed based on R-Studio for performing bibliometric analysis (8). It provides data processing and visualization functions, and supports importing literature data from databases such as Web of Science and PubMed, and performing multidimensional analysis on data of literature, providing readers with an objective view of distribution of global research, frontiers, major potential problems and thematic trends in a specific field (9). BiblioShiny is a graphical user interface of Bibliometrix developed in recent years, which facilitates users in completing complex analyses.

Scientometric studies have been carried out on certain Covid-19 relevant research fields, such as the COVID-19 research productivity of the United Arab Emirates (UAE)-affiliated researchers (10), the research on COVID-19, SARS-CoV-2 in dermatology journals (11), the intersection of nanotechnology and SARS-CoV-2/COVID-19 (12), the scientific publications on drugs and therapies used to treat COVID-19 (13), etc. However, to date, there is a lack of comprehensive scientometric analysis of the association between SARS-CoV-2 and the reproductive system. Ascertainment of the distribution situation, changing patterns, major shortness, thematic trend, and global cooperation nets, etc, of the research in this field can help formulate future research and scientific resource investment strategies.

Thus, this study retrieved articles and reviews on association of SARS-CoV-2 with genital cells from the Web of Science Core Collection (WOSCC), Scopus and PubMed databases, and performed comprehensive scientometric analysis of the literature regarding this viral infection and the human genital system to identify the knowledge structure, key hotspots, thematic trends, and major issues in this field, with the aim of guiding future research directions and fostering collaboration.

## 2 Materials and methods

### 2.1 Data sources

The data used for the scientometric analysis were retrieved from the WOSCC, Scopus, and PubMed databases, which are public databases that extensively collects scientific data in various fields. The search formula used was (Topic: COVID-19 OR SARS-CoV-2) and (Topic: reproductive OR reproduce OR reproduction OR genital) and (Topic: cell OR cells OR cellular). The search period was limited to that of publication from January 1, 2020 to December 31, 2025, while the types of documents were limited to articles and reviews.

### 2.2 Data inclusion

The documents were retrieved and identified by Linghan Zhang and Minkai Wang. respectively, according to the same criteria, and then cross-checked by them. Inclusion criteria: Among the retrieved documents, only original articles and reviews formally published or in early access were included. Exclusion criteria: Editorial materials, meeting abstracts, preprints, video-audio media and letters were excluded. The data from the three databases were merged using Biblioshiny 5.0, and used for the following analysis.

### 2.3 Data analysis and visualization

Bibliometrix/BiblioShiny 5.0 and WPS Office/EXCEL365 were used in this study to analyze the distribution of the literature with publication date from 2020 to 2025 by countries, journals, references, authors, citations and keywords, etc., and to display the impact and importance of the documents, journals, authors, affiliations and countries, trend of publication, citation over the years, thematic trend, collaborations among nations, affiliations, etc. BiblioShiny was also used to visualize the analysis results. All the primary data used here were obtained via searching the public databases, and thus did not require ethical review.

## 3 Results

### 3.1 The publication outputs trend

The study identified 511 documents from WOSCC, 1,654 items of literature from Scopus and 1,017 documents from PubMed. Through merging the collections using BiblioShiny, totaled 1,802 documents were included in the WOSCC-Scopus (WS) dataset with deletion of 357 duplicated ones; and a total of 2,354 records were included in the WOSCC-Scopus-PubMed (WSP) dataset with deletion of 817 duplicated ones, 9 preprints, 1 abstract and 1 video-audio media.

The literature in the WSP dataset was contributed by 13,365 authors, and published in 1,054 journals and 3 books. The National Natural Science Foundation of China (181 records), the National Institutes of Health (68 records), the UK Research and Innovation (46), and National Science Foundation(USA) (45 records), National Key Research and Development Program of China (39 records) were the most frequent funding sponsors, and 1,380 documents were published in open-access journals, according to the 1,654 records from WOSCC.

**Figure 1:**
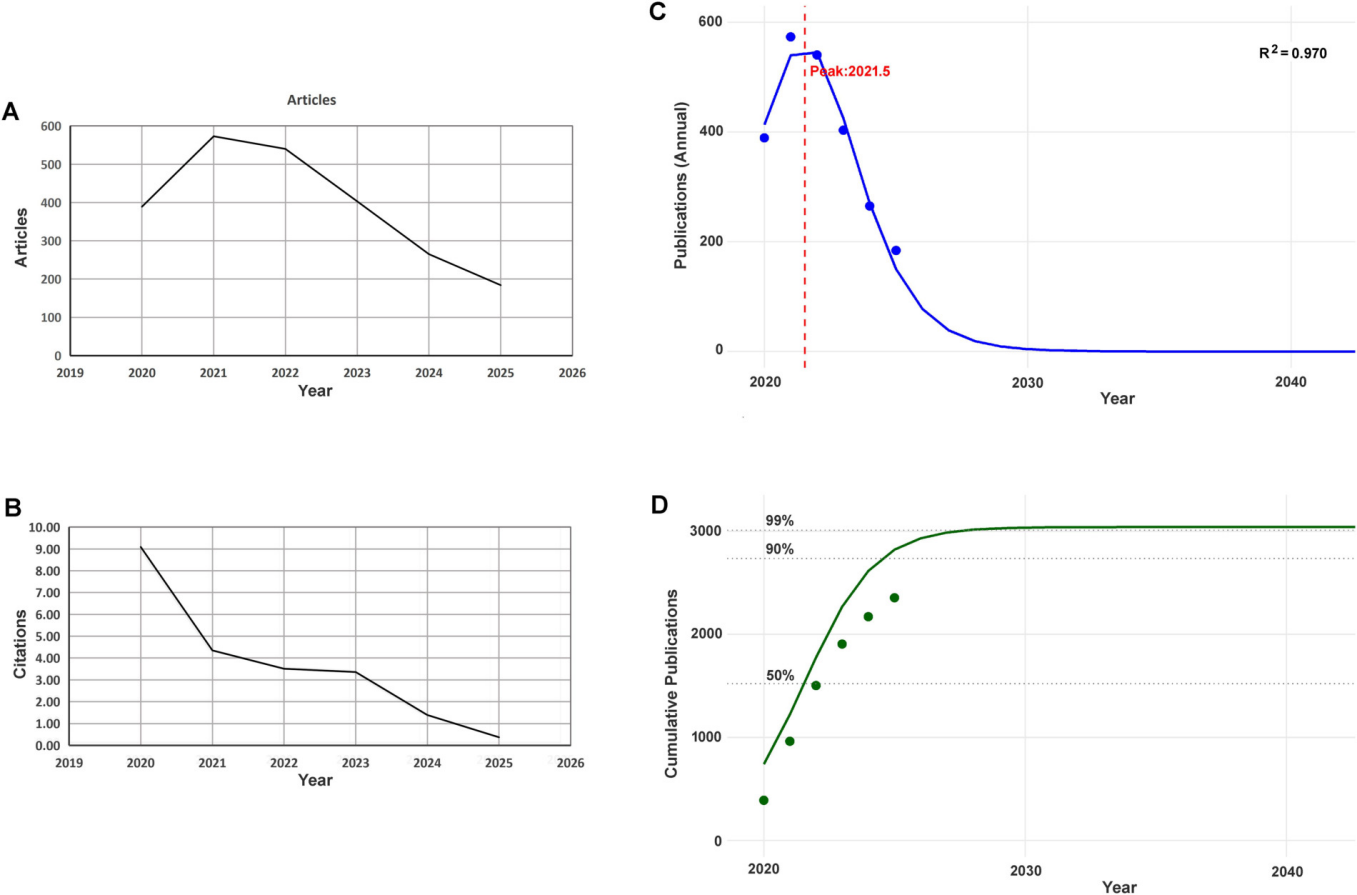
Scientific production, citations and the life cycle and growth curve of annual publications. **(A)** Annual scientific production trend; **(B)** Average citations per year; **(C)** Life cycle of annual publications; **(D)** Cumulative growth curve of annual publications.

The annual scientific production of the publications on the association of SARS-CoV-2 with genital system rapidly increased from 389 in 2020 to 573 articles in 2021, slowly decreased from 2021 to 2022, and then quickly went down year by year to 184 in 2025 (Figure 1A). Average citations per year (MeanTCperYear) of the publications decreased from 9.09 citations in 2020 to 3.36 citations in 2023, and then dropped to 1.39 citations in 2024 and 0.37 citations in 2025 (Figure 1B).

A Life cycle analysis of annual publication resulted in that the annual publication curve of the documents progressed to the top in May, 2021, and then sharply declined, and will be near to zero from 2030 onward (Figure 1C). The cumulative growth curve of annual publication shows the process and trend of the cumulative publications (Figure 1D).

**Figure 2:**
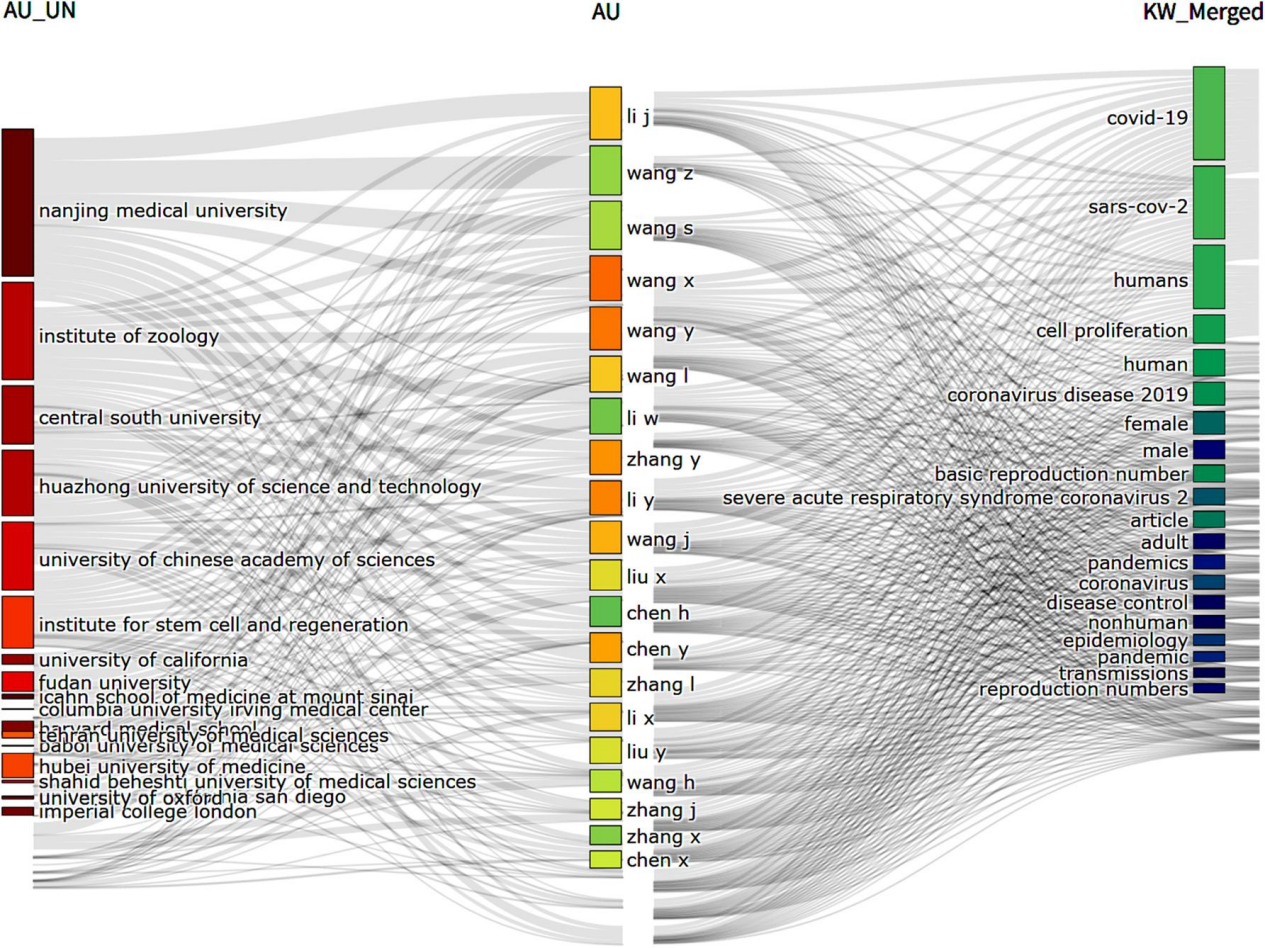
Sankey diagram plotted with biblioShiny. The diagram used the affiliations (AU_UN), authors (AU) and keywords (KW) as the three fields, showing the impactful authors and their affiliations and frequent research keywords.

A three-field plot (namely Sankey diagram) of the relevant literature was drawn with BiblioShiny, using Affiliations (AU_UN), Author (AU) and Keywords (DE) as the three fields. The Sankey diagram illustrates relative importance of the involved items in the same field, showing Li J., Wang Z., Wang S., Wang X., et al. as the influential authors, and Nanjing Medical University, Institute of Zoology, University of Chinese Academy of Sciences, Huazhong University of Science and Technology, Central South University, etc. as the affiliations of the influential authors (Figure 2).

### 3.2 Sources and authors of the literature

As results of the analysis on the sources of the literature, the most relevant sources and amount of relevant publications are shown in Supplementary Table S1. The local impact of the journals is shown in Supplementary Figure S1. The most relevant authors of the literature were Wang X, Wang Y, Li Y, Zhang Y, et al. The authors with high local impact are shown in Supplementary Table S2. The most relevant affiliations are shown in Supplementary Figure S2.

**Figure 3:**
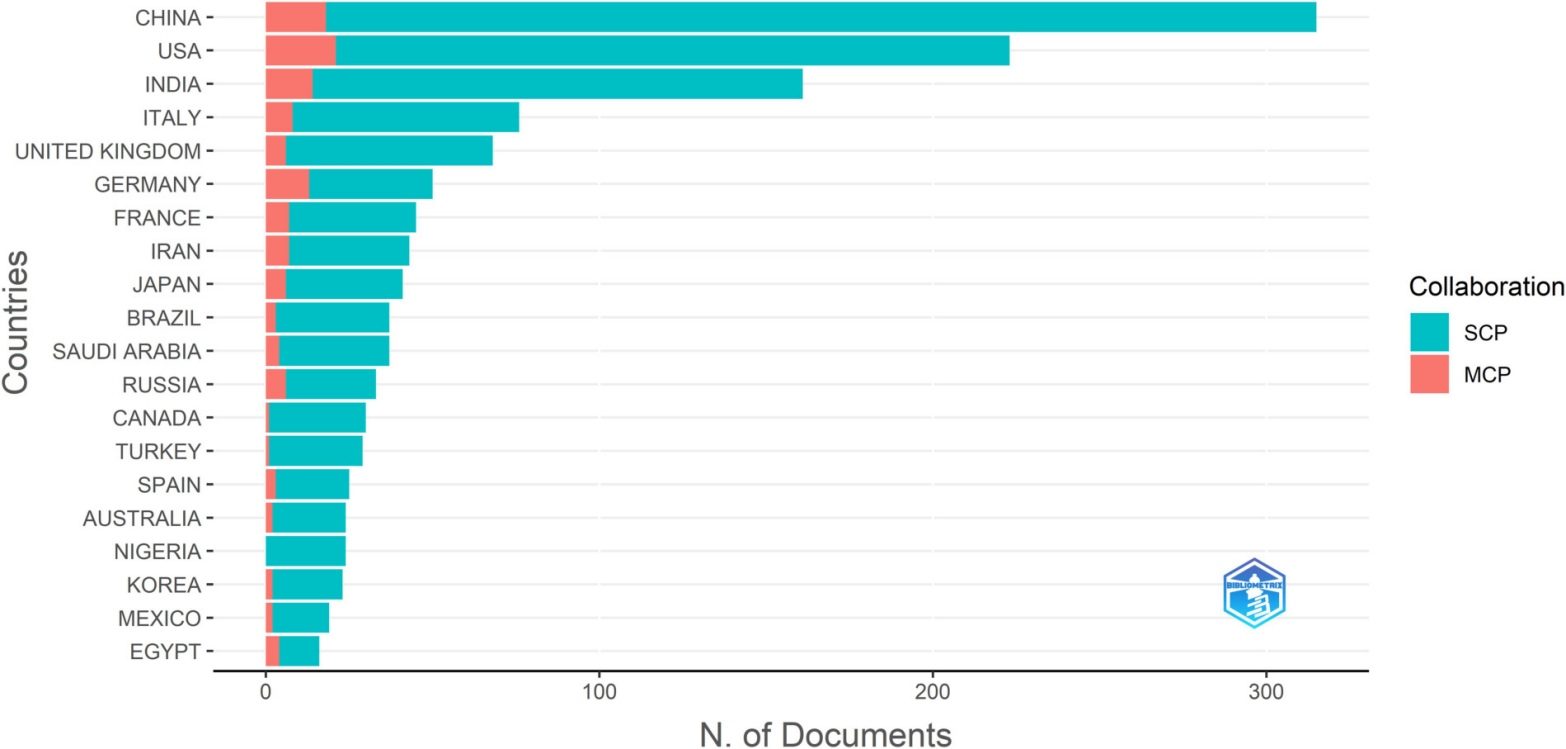
Corresponding authors’ countries by quantity of the documents (based on the WOSCC-scopus datasets). SCP, Single Country Publications. MCP, Multiple Country Publications.

As corresponding author's countries, China and USA had much higher production of relevant documents than other countries (Figure 3). However, Sweden and Portugal had the highest average article citations among all the corresponding author's countries (Table 1).

**Table 1:** Top countries by their authors’ citations (based on the WOSCC-scopus dataset).

Country	Total Citation	Average Article Citations
USA	12,847	57.60
CHINA	10,108	32.10
UNITED KINGDOM	5,009	73.70
INDIA	4,226	26.20
ITALY	2,047	26.90
IRAN	1,419	33.00
GERMANY	1,305	26.10
JAPAN	1,082	26.40
NIGERIA	1,015	42.30
FRANCE	925	20.60
SWEDEN	789	131.50
SAUDI ARABIA	785	21.20
CANADA	693	23.10
INDONESIA	618	51.50
TURKEY	616	21.20
PORTUGAL	612	122.40

### 3.3 Documents and keywords

**Figure 4:**
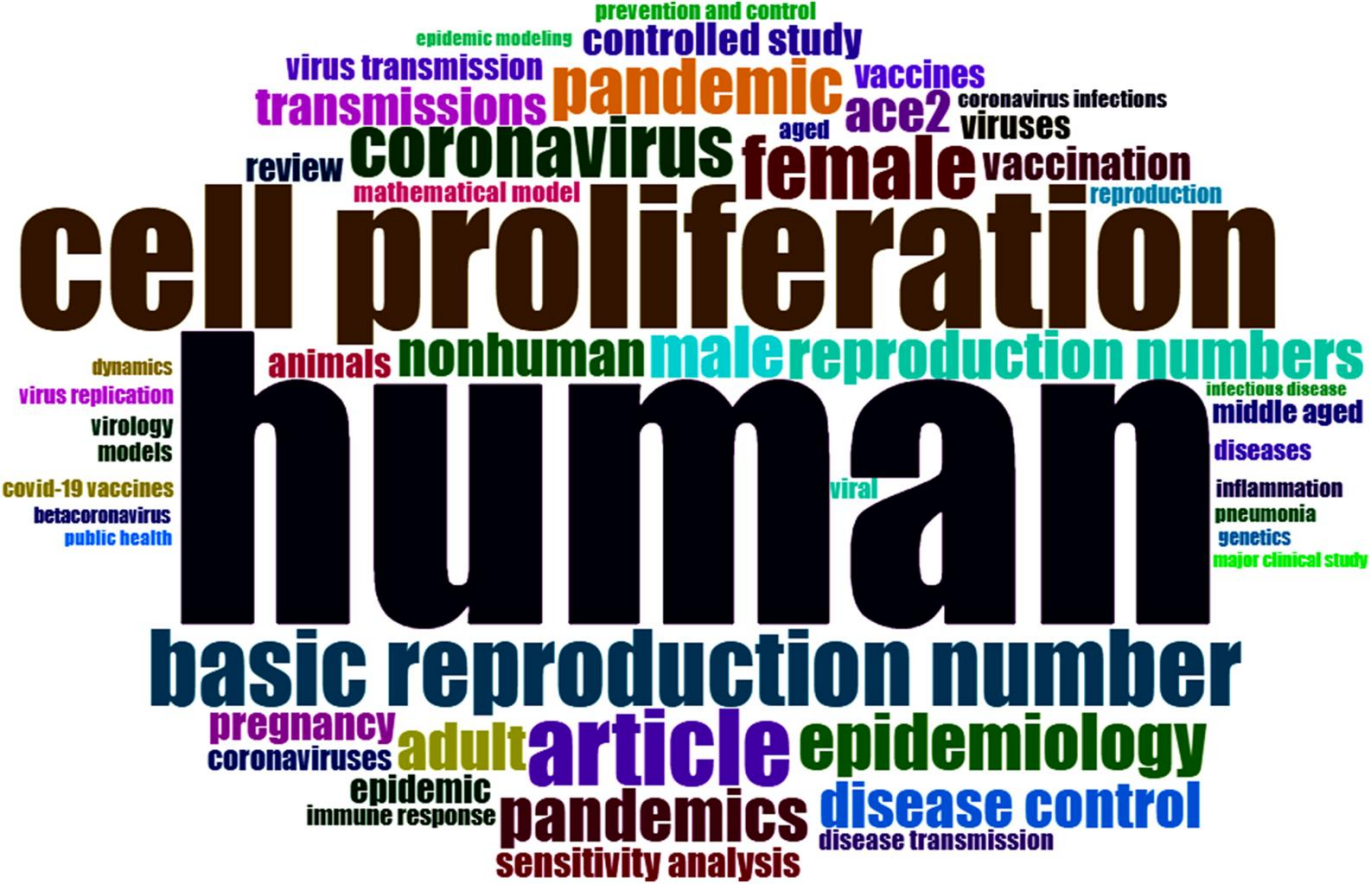
Wordcloud of the frequent keywords excluding COVID-19 and SARS-CoV-2. The figure indicates that human, cell proliferation and basic reproduction number were the keywords with highest frequency, excluding COVID-19 and SARS-CoV-2.

**Figure 5:**
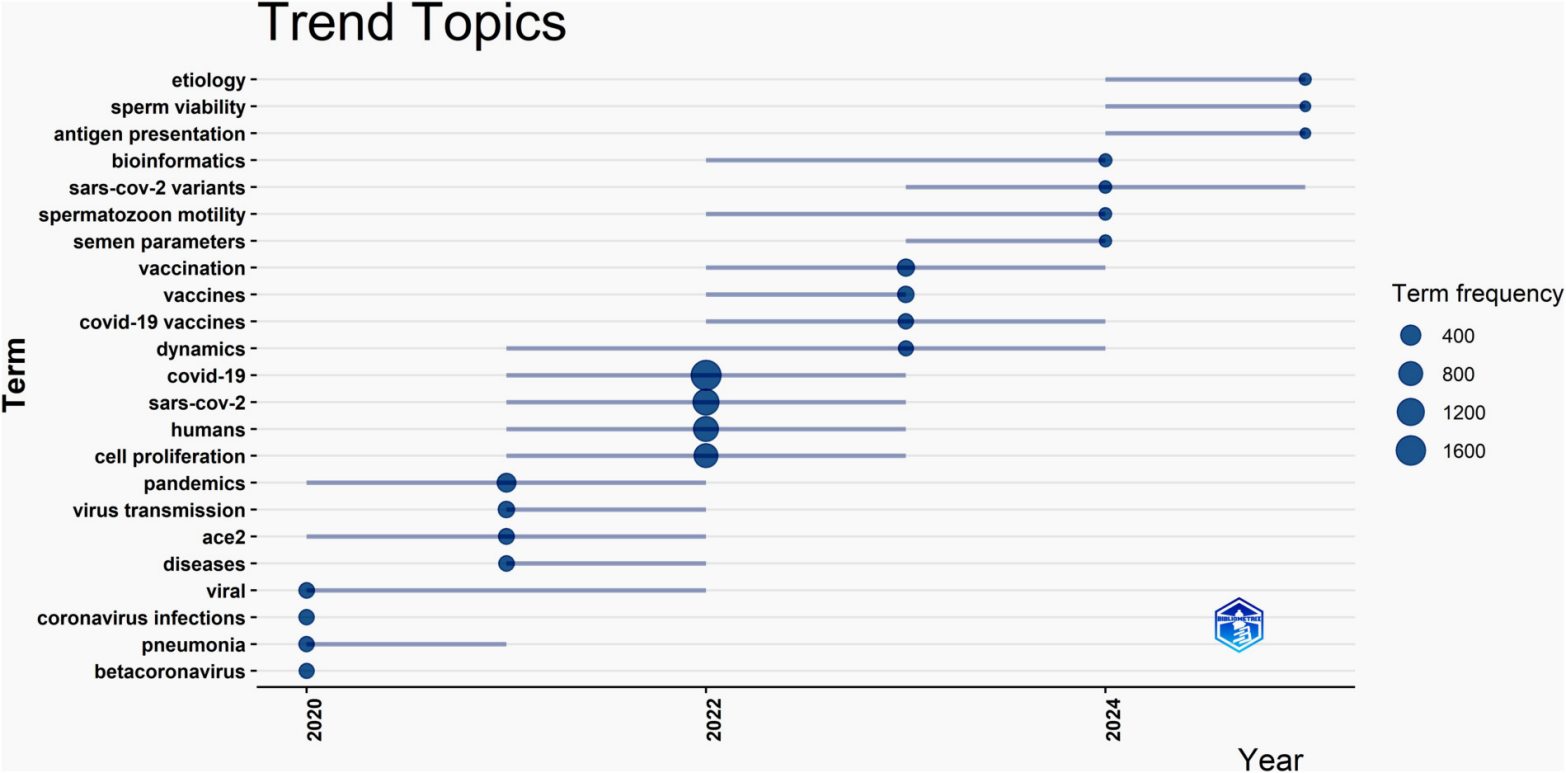
Trend topics of the relevant research by years. The figure shows the changes in trend topics in this field, with etiology, antigen presentation, and sperm viability emerging as newly prominent topics in 2025.

Among the documents included in this study, the most global cited reports are shown in Supplementary Figure S3. The most influential documents were those by Fajgenbaum D (30), Ferretti L (31), Plante J (32), et al., respectively. The most frequent words were covid-19, human, sars-cov-2, cell proliferation, pandemic, basic reproduction number, etc. Figure 4 shows a Wordcloud drawn using Biblioshiny with 50 most frequent keywords excluding COVID-19 and SARS-Cov-2. Figure 5 shows trend topics in this research field by year.

### 3.4 Comprehensive analysis

**Figure 6:**
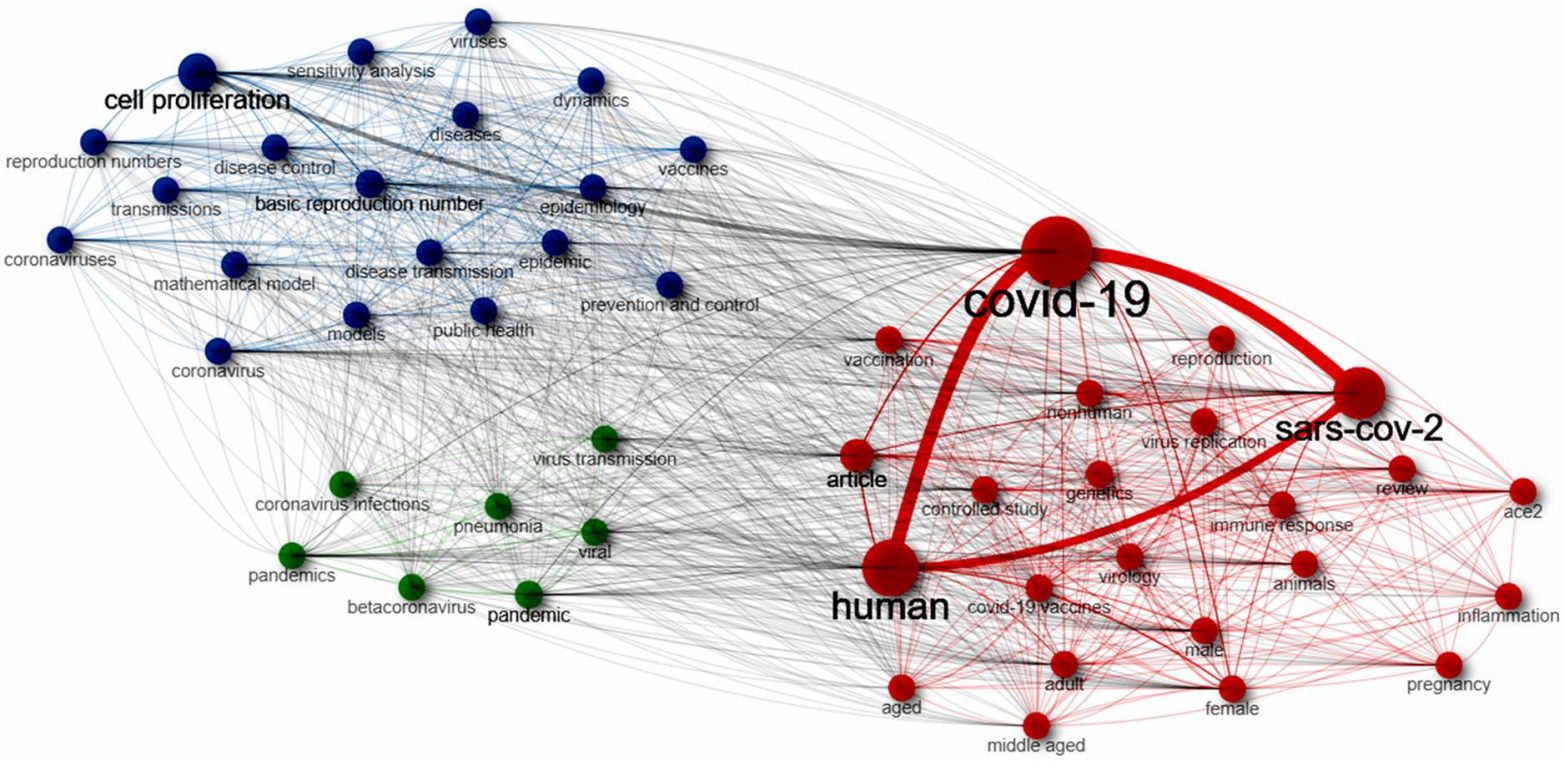
Co-occurrence network formed on the keywords from the documents showing research hotspots in this field.

A co-occurrence network was formed based on the keywords of the documents (Figure 6). There are three clusters in the network. The cluster 1 includes covid-19, human, sars-cov-2, female, male, adult, nonhuman, ace2, vaccination, pregnancy, controlled study, animals, review, middle aged, immune response, etc. The cluster 2 comprises cell proliferation, basic reproduction number, coronavirus, epidemiology, reproduction numbers, disease control, transmissions, sensitivity analysis, epidemic, mathematical model, etc.

**Figure 7:**
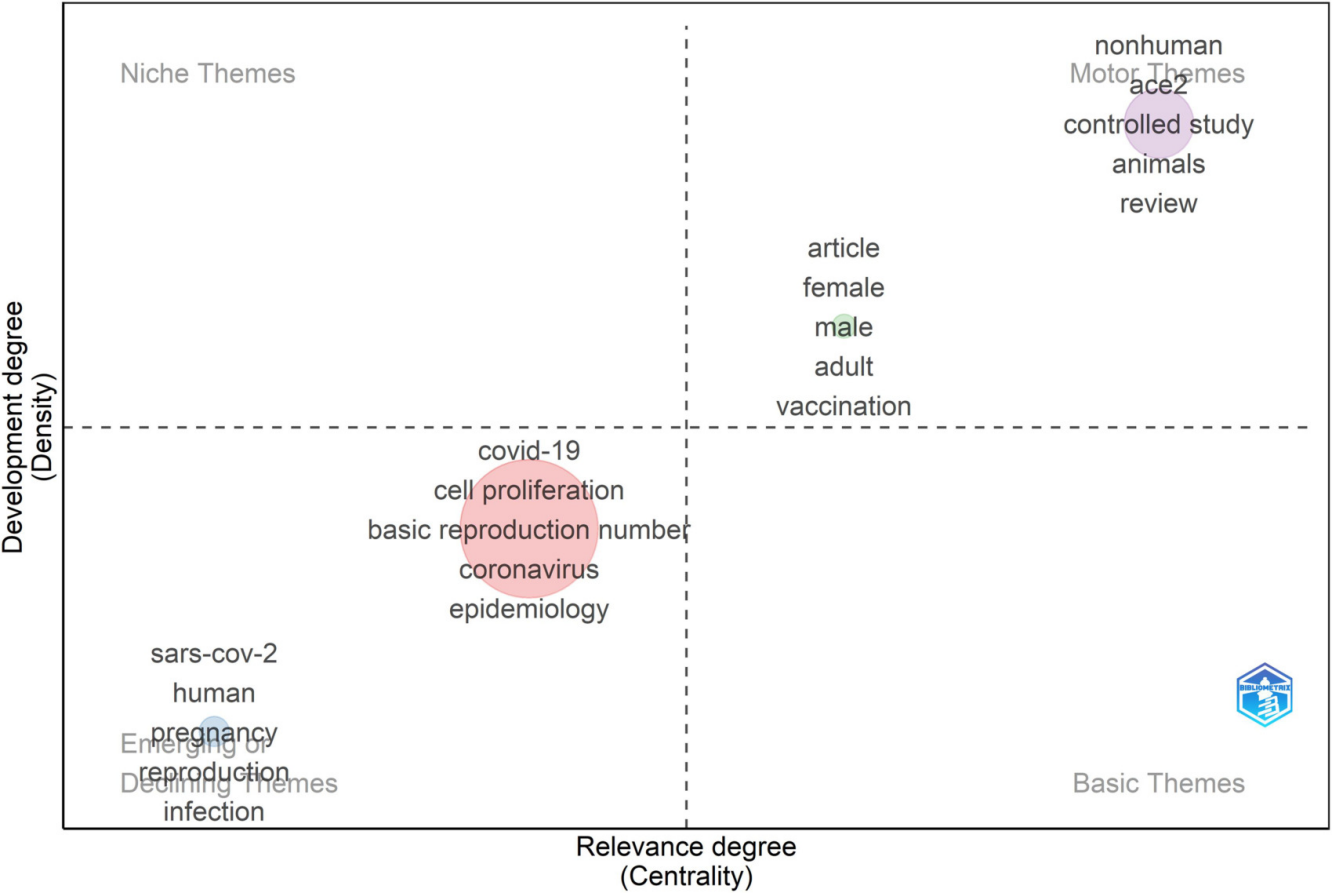
Thematic map of the research field showing theme centrality and development level.

Using a thematic map, the development degree and relevance degree of four clusters of the keywords are shown in Figure 7. The clusters are labeled with the keywords of highest occurrences in the clusters. The cluster “sars-cov-2” includes human, sars-cov-2, pregnancy, reproduction, infection, cells, expression, fertility, semen, male infertility, testis, infertility, spermatogenesis, oxidative stress, tmprss2, pregnancy complications, infectious, male fertility, reproductive health, testosterone, etc., locating in the lower left quadrant. By factorial analysis using the method of multiple correspondence analysis, 50 of the keywords from the included document are sorted into three clusters, as shown in the conceptual structure map (Supplementary Figure S4).

The collaboration network by the affiliations in this field is shown in Supplementary Figure S5, and the affiliations with collaboration are divided into eight groups. Among the affiliations, Kyoto University, Hokkaido University, and Egyptian Knowledge Bank (EKB), had highest impact (PageRank) in the first group. Imperial College London, University College London, and University of Oxford in the second group, Chinese Academy of Sciences, Fudan University, and Peking University in the third group, and INSERM, Universite Paris Cite and Assistance Publique Hopitaux Paris (APHP) in the fourth group had the highest impact in their groups. The Supplementary Figure S6 shows the USA, China, Italy, Germany, India conducted more collaborations than other countries.

## 4 Discussion

### 4.1 General information

During the COVID-19 pandemic, global scientific resources were heavily invested in research on the viral infection and pathogenesis, clinical management, epidemiological factors, and prevention of COVID-19, yielding a vast body of literature (14–16). The results of this study show that the annual output of relevant research reports can be divided into three stages based on the trend in quantity of publications: In the first stage, from 2020 to 2021, a rapid increase in the research reports appeared; in the second stage, from 2021 to 2022, the annual publication volume slightly changed, presenting a platform phase; in the third stage, from 2022 to 2025, the annual publication rapidly declined. The possible factors might include: Firstly, In the early phrase of the epidemic, approximately from the end of 2019 to 2021, the COVID-19 outbreak and the serious harm of the early highly pathogenic viral strain to human health attracted global attention, resulting in rapid and enlarged investment of health resources in the research on COVID-19. Furtherly, the discovery of viral receptors in the male reproductive system might play an important role in the fast growth of the related research (17, 18); secondly, from 2021 to 2022, the global pandemic situation, the diversified pathogenicity of the mutant strains and, specifically, detection of the viral nucleic acids in semen contributed to keep high input and output levels of the relevant research (4, 19); thirdly, from 2022 on, with the further decline of the pathogenicity of the virus strain, the easing of the global pandemic and the weakening of the prevention and control pressure, the research on COVID-19 rapidly subsided, which also affected the studies of the relationship between SARS-CoV-2 infection and reproductive health.

The life cycle analysis of annual publication resulted in that the annual publication started rising in 2020, reached to the top in 2021, then rapid declined until 2025, and was predicted to decrease near to zero in 2030 (Figure 1C). The cumulative growth curve of annual publication showed the cumulative publications had occupied 90% of the literature saturation volume in 2025 (Figure 1D). The findings indicate that the topic of the included research has developed into the maturity phase (50%–90% of saturation), and thus its growth had been slowing. As predicted, from 2030 onward, almost no cumulative growth will exist, namely the annual publication of the relevant studies will be near to zero. These data indicate potential serious problem on development of the relevant research, which will be discussed below.

The Sankey diagram (Figure 2) shows the impactful authors in this field and their affiliations. Among them, the scholars from the Nanjiing Medical University, Chinese Academy of Sciences (like Institute of Zoology), Central South University, and Huazhong University of Science and Technology exhibited high impact in this field. The right field show the keywords related to the studies which these influential authors conducted. However, almost no keywords closely related to the impact of this infection or disease on genital cells are listed here, indicating shortness of further investigation into this field.

### 4.2 Sources and authors of the literature

The relevant journals belongs to the disciplines of medicine, mathematics, biochemistry, genetics and molecular biology, engineering, computer science, immunology and microbiology, agricultural and biological sciences, physics and astronomy, environmental science, pharmacology, toxicology and pharmaceutics, chemistry, chemical engineering, materials science, neuroscience, multidisciplinary, etc. The findings demonstrate interdisciplinary collaboration for prevention and control of this emerging infectious disease, supporting the One Health concept in this field. In addition, most of them are open-access journals.

Among the authors' countries, China, USA, India, Italy, UK, and Germany had larger quantities of the documents than others (Figure 3). However, Sweden had the highest average article citation (131.50), much more than UK (73.70), USA (57.60), China (32.10), Japan (26.40), etc. These data suggest that improvement of quality might be more important than enhancement of the quantity of the production in certain countries.

### 4.3 Documents and keywords

The most global cited documents were “Cytokine Storm” (30), “Quantifying SARS-CoV-2 transmission suggests epidemic control with digital contact tracing”(31), and “Spike mutation D614G alters SARS-CoV-2 fitness”(32), respectively, indicating the highest influence of these literature. The most frequent words were human, cell proliferation and basic reproduction number besides covid-19 and sars-cov-2 but almost no keywords closed to the genital cells. This result is basically in accordance with the data shown in the Sankey diagram, indicating the impact of this viral infection on genital cells or fertility was not the major hotspots for most time in the period.

The analysis of trend topics showed that the main hot topics were kept changing over the years, for instance, they were viral, pneumonia and coronavirus infection in 2020, pandemics, virus transmission, ace 2 and diseases in 2021, COVID-19, cell proliferation and SARS-Cov-2 in 2022, vaccination, vaccines, and dynamics in 2023, bioinformatics, sars-cov-2 variants, spermatozoon motility and semen parameters in 2024, etiology, antigen presentation and sperm viability in 2025. These data indicates that the effect of this viral infection on human fertility are newly emerging trend topics, indicating that they attracted research attention later than the others. Moreover, research has focused more on the impact of this virus on male fertility rather than on female reproduction.

### 4.4 Comprehensive analysis

The co-occurrence network (Figure 6) shows three main clusters of the keywords. Among these keywords, almost all those related to the impact of covid-19 on reproductive system, such as male, female, pregnancy, are in the first group. However, none of the keywords on pathogenesis of genital cells at cellular or molecular levels, such cell signaling pathways, omics, gene expression profiles, are included, suggesting the association between SARS-Cov-2 and human reproduction might be seriously neglected by most research.

The thematic map (Figure 7) displays the development level and relevance degree of four clusters of the keywords. The most influential cluster represents the thematic topic related to the COVID-19, cell proliferation, basic reproduction number, coronavirus, epidemiology, and pandemic, etc. The cluster “sars-cov-2” includes the keywords, such as human, sars-cov-2, pregnancy, reproduction, infection, cells, expression, fertility, semen, male infertility, testis, infertility, spermatogenesis, oxidative stress, tmprss2, pregnancy complications, infectious, male fertility, reproductive health, testosterone, etcshow thematic topics, showing thematic topic closely linked to the relationship between the viral infection and human fertility. However, this cluster is located in the lower left quadrant, suggesting early appeared but underdeveloped topics (20). This result might be due to the insignificant importance of these themes or a long-term neglect of them.

The factorial analysis gave insight into closeness among the keywords and the clusters, indicating potential factors for clustering of the terms. The results of the factorial analysis showed relationship between the keywords, basically in accordance with the findings of the co-occurrence network analysis (Supplementary Figure S4).

The collaboration networks plotted by the affiliations and countries of the authors, respectively, show more than 90% of the collaborations occurred among the affiliations in less than 10% of the global countries. Most African, Latin American, and Asian countries rarely had international research cooperations. Among the author affiliations, Kyoto University, Imperial College London, Chinese Academy of Sciences, INSERM, exhibited highest impact in the groups of international collaborations. These data showed the markedly uneven development of scientific research in this area, which might cause considerable problems for global control of the long-term impact of this emerging infectious disease on mankind (21).

### 4.5 Problems in the research area

First of all, accumulated evidence shows that human multiple systems can be involved in COVID-19 (22, 23), the recent emergence of the long Covid indicates the complexity of the interaction and underlying mechanism between COVID-19 and human, and the long-term effect of this viral infection, especially, that on the human fertility, is far from clear, but can be of significant importance for public health safety (24, 25). Nevertheless, the data indicate that, firstly, with the easing of the COVID-19 epidemic, the global research on the effect of this infection on human reproductive health had declined rapidly, and was predicted to be almost in stagnation by 2030; secondly, compared with the male reproductive system, the literature on the viral impact on the female fertility was much less, which might be related to the detection of viral nucleic acid in semen. However, there is no evidence that the pathogenic effect of SARS-CoV-2 on genital cells depends on presence of viral nucleic acid; thirdly, there was a significant imbalance among global countries in distribution of the publications, the high-yield and influential authors and affiliations, and international collaboration in this field, which could be detrimental to the development of research in this field, and should attract global enough attention.

### 4.6 Suggestion for future efforts

Since the sources, health impact and trend of SARS-CoV-2 infection still remain undefined (26, 27), it might be irrational with high-risk to acquiesce that this virus is similar to the well-known naturally originated viruses in pathogenesis, and the influence of its infection will disappear as the pandemic subsides. In future, at the macro level, cohort studies should be strengthened focusing on the long-term impact of SARS-CoV-2 infection on the infected population and their offspring (4, 28, 29); at the micro level, the genetic characteristics and variation patterns of this virus, the role of viral nucleic acids and virulence factors on the stability and genetics of human genital cell genomes, potential intervention drugs and targets for the viral infection of germ cells should further approached, utilizing bioinformatics, multi-omics analysis, and molecular epidemiological techniques. Meanwhile, the research on COVID-19 impact on female fertility should be paid enough attention, and the relevant trend topics on etiology, antigen presentation, and sperm viability should also be promoted. In addition, more global efforts should be made for promoting international cooperation, balancing the allocation of scientific resources and development of research in this field.

### 4.7 Strength and limitations

This study performed comprehensive scientometric analysis on 2,354 items of literature (2020–2025) from WOSCC, Scopus, and PubMed databases, revealing the development status, impactful authors, journals, affiliations, hotspots, trend topics, collaborations in the research regarding the impact of SARS-CoV-2 infection on human reproductive system. Especially the report highlights the major issues in this field, including long term and seriously insufficient efforts on deeply investigate the interaction and molecular mechanism between this newly emerging viral infection on human genital cells, particularly on female reproduction, and the global uneven development of the research in this field.

Our study has limitations.When merging the data from the three databases, WOSCC, Scopus, and PubMed, the study encountered the database compatibility issues. The study constructed two datasets, WS and WSP, and used the WS when the WSP triggered functional failures in the analysis software. Therefore, a small portion of the analysis was based on the data from only two databases, and the resulting figures and tables have been labeled in the report. In addition, the limitations includes the inability to discuss with certainty the language bias, the indexing pathways, the temporal limitations (2020–2025), and the determinations derived from the scientometric analysis.

## 5 Conclusion

This study is the first time to perform comprehensive scientometric analysis on the literature (2020–2025) regarding the association of SARS-CoV-2 with reproductive system by using bibliometrics/BiblioShiny, revealing the overall distribution of the publications across time, authors, countries, institutions, journals, and the hotspots, frontiers, and thematic trends, as well as landmark studies, highly productive and influential authors, institutions, and collaboration networks. Furtherly, the study identifies the major problems on the research distribution, progression trend and collaborations in this field. The report offers the latest macroscopic perspectives, references, and clues for formulating research strategies, management of public health resources, and efficient collaborations in this scientific area.

## Data Availability

The original contributions presented in the study are included in the article/Supplementary Material, further inquiries can be directed to the corresponding author/s.
