# Supplementary material for: SARS-CoV-2 and reproductive system: a scientometric study
Source: Front Reprod Health. 2026 May 14;8:1844245. doi: 10.3389/frph.2026.1844245 (PMC13216177; doi:10.3389/frph.2026.1844245)
Supplement: Supplementary file 1 [file Datasheet1.pdf]

Supplementary Material

1 Supplementary Figures and Tables

1.1 Supplementary Figures

Supplementary Figure 1. Sources' local impact of the literature by H index.

Supplementary Figure 2. Most relevant affiliations of the authors (Based on the WOSCC-Scopus dataset).

Supplementary Figure 3. Most global cited documents, showing literature with high citations.

Supplementary Figure 4. Conceptual structure map of the frequent keywords by factorial analysis.

Supplementary Figure 5. Collaboration network of the authors' affiliations in the field (Based on the WOSCC-Scopus dataset).

Supplementary Figure S6. Countries' collaboration world map of the research area (2020–2025).

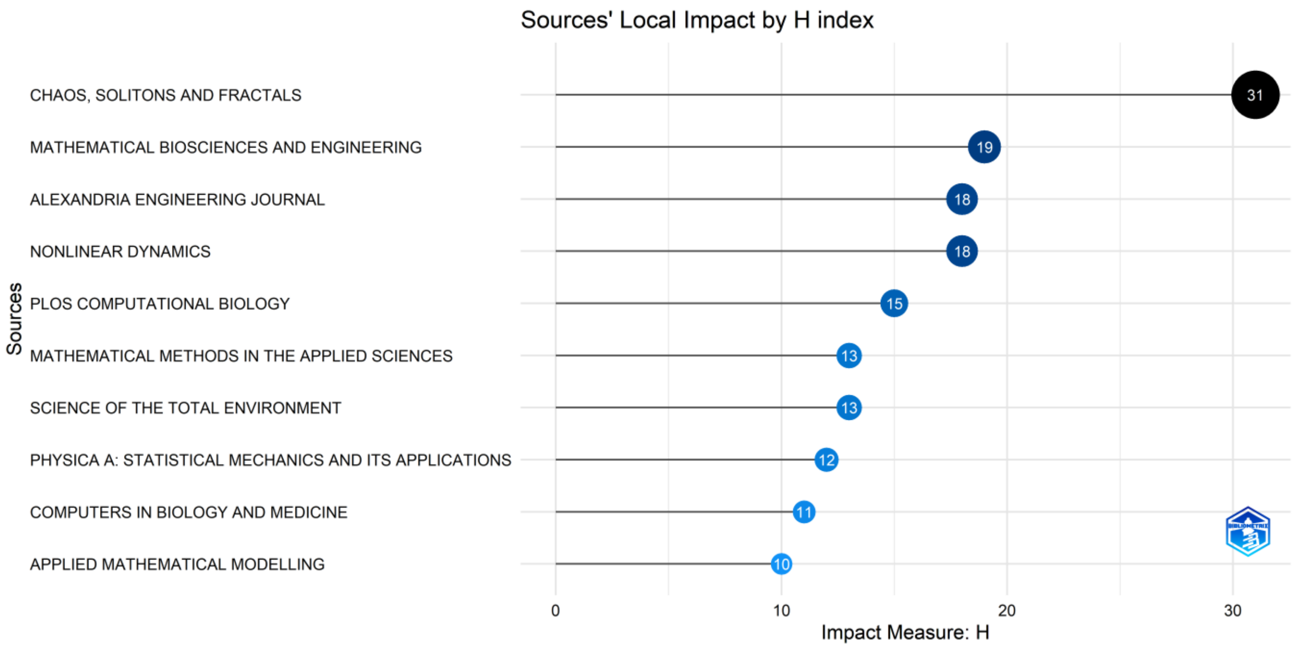

Supplementary Figure 1. Sources' local impact of the literature by H index.

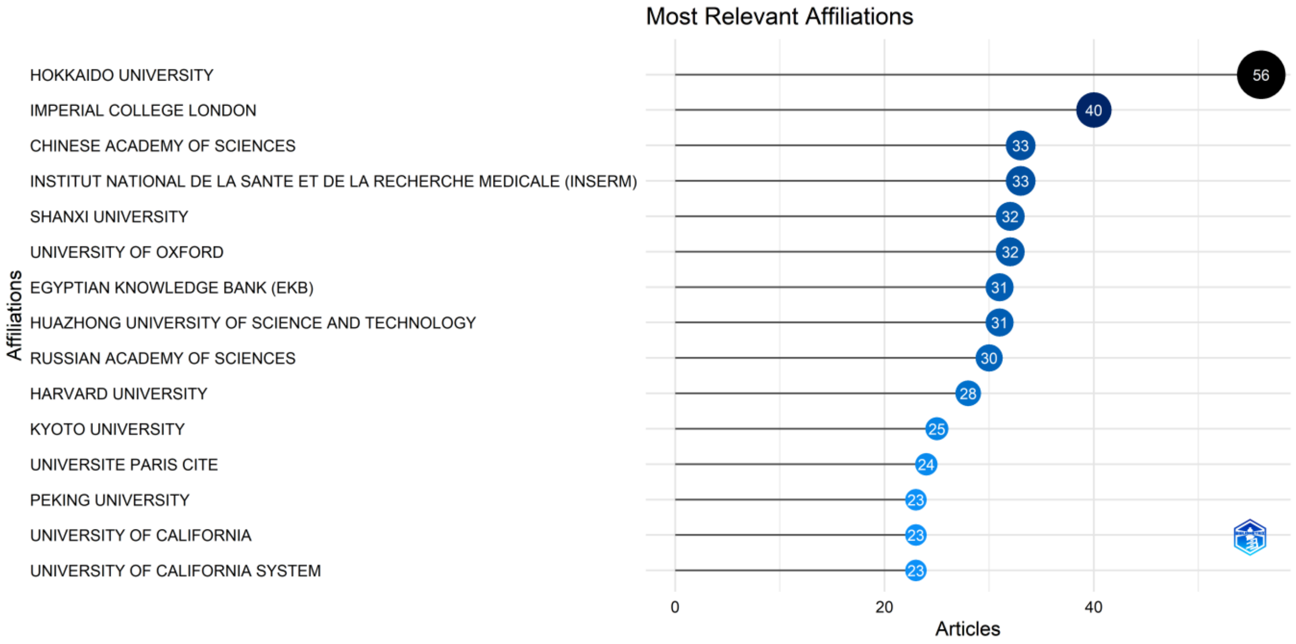

Supplementary Figure 2. Most relevant affiliations of the authors (Based on the WOSCC-Scopus dataset).

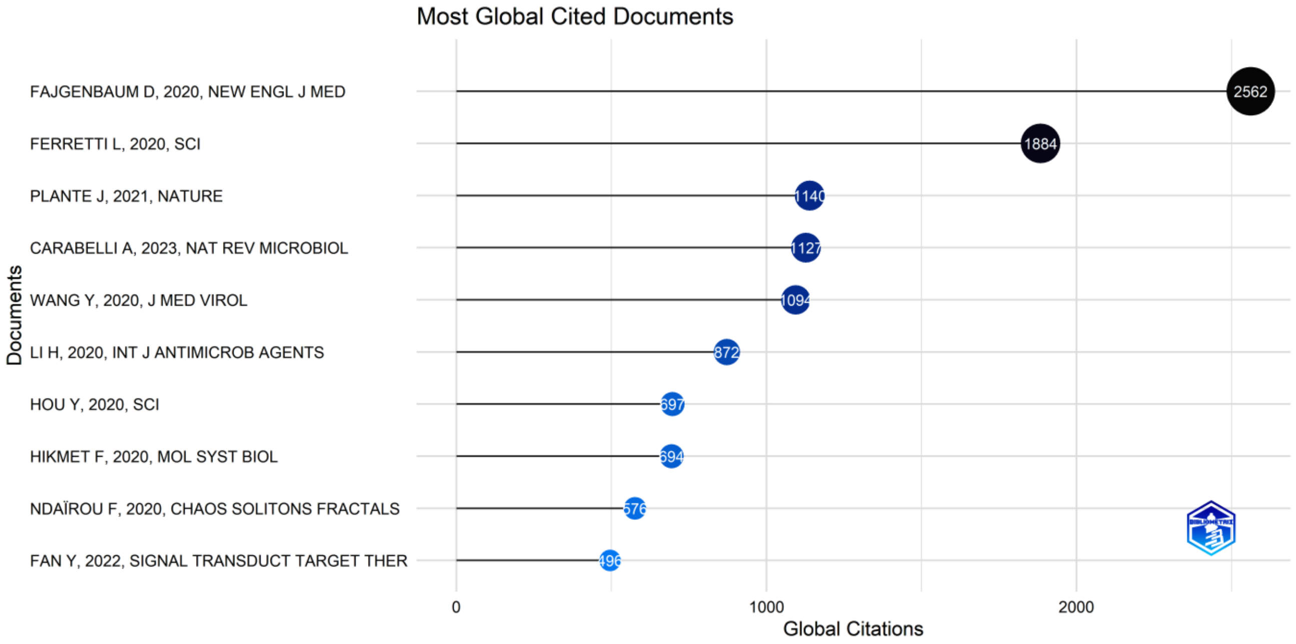

Supplementary Figure 3. Most global cited documents, showing literature with high citations.

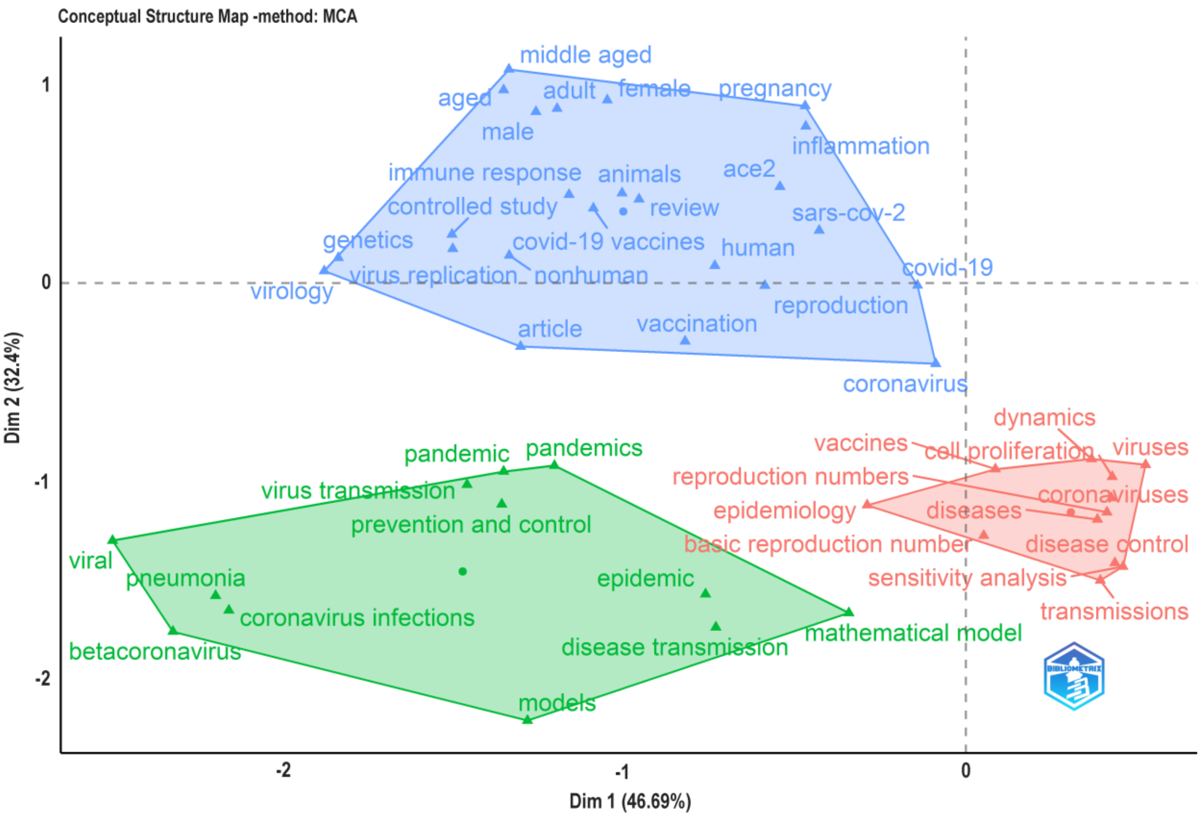

Supplementary Figure 4. Conceptual structure map of the frequent keywords by factorial analysis.

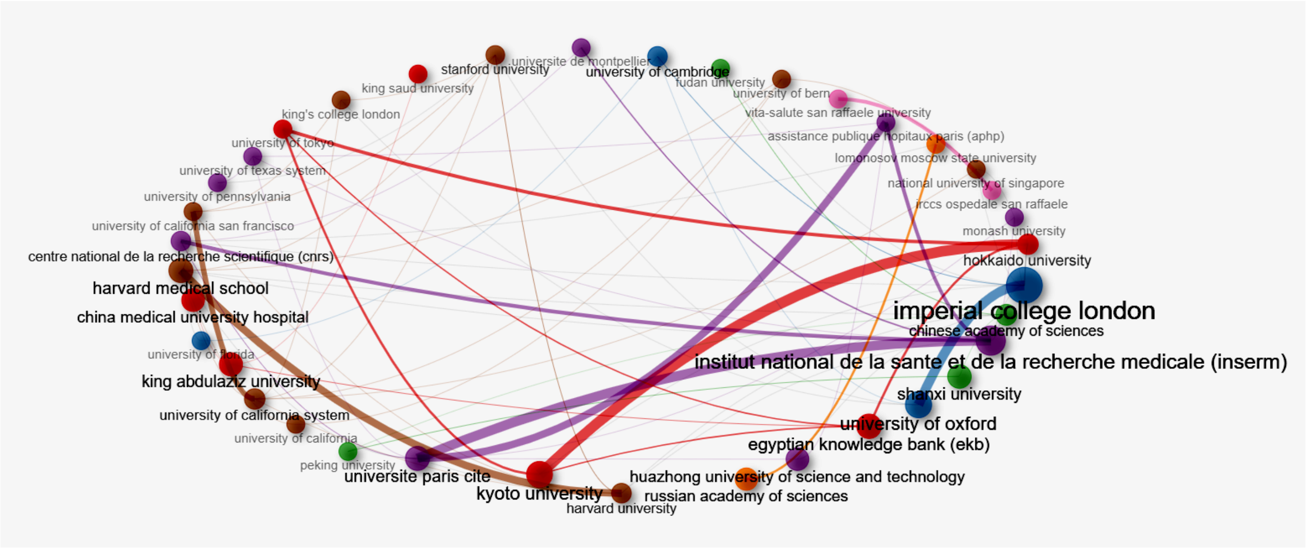

Supplementary Figure 5. Collaboration network of the authors’ affiliations in the field (Based on the WOSCC-Scopus dataset).

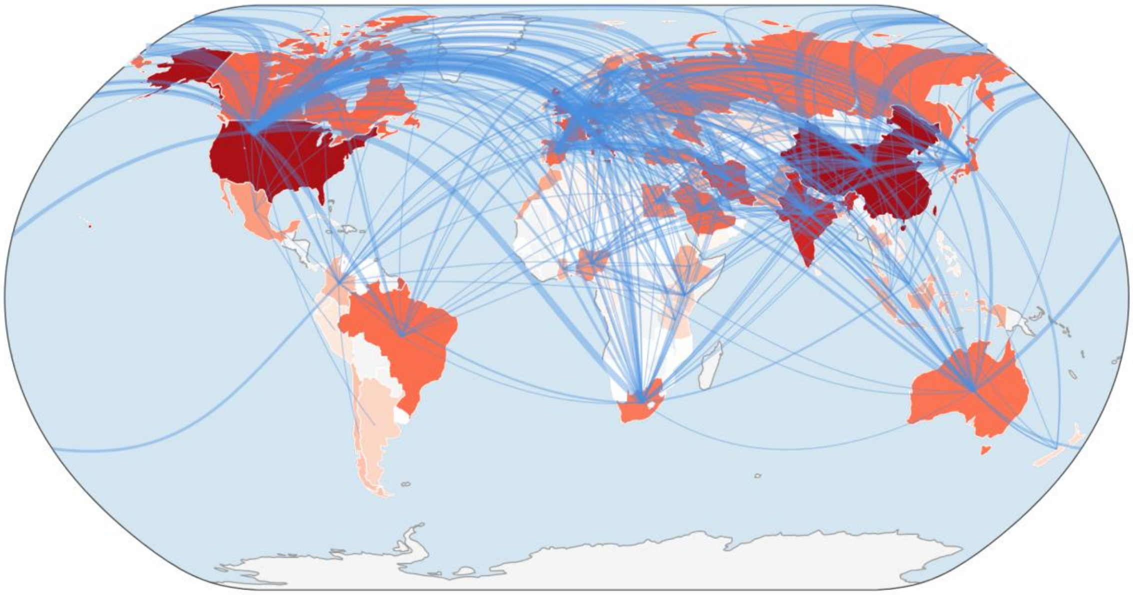

Supplementary Figure 6. Countries' collaboration world map of the research area (2020 – 2025).

2.2 Supplementary Tables

Supplementary Table 1. Most relevant journals and their publication counts.

Supplementary Table 2. Authors' local impact measured by six indicators.

Supplementary Table 1 Most relevant sources of the literature and their publication counts.

Sources	Articles
MATHEMATICAL BIOSCIENCES AND ENGINEERING	69
CHAOS, SOLITONS AND FRACTALS	66
NONLINEAR DYNAMICS	45
PLOS COMPUTATIONAL BIOLOGY	44
FRONTIERS IN IMMUNOLOGY	41
INTERNATIONAL JOURNAL OF MOLECULAR SCIENCES	31
ALEXANDRIA ENGINEERING JOURNAL	27
MATHEMATICAL METHODS IN THE APPLIED SCIENCES	27
PLOS ONE	18
SCIENTIFIC REPORTS	18
VIRUSES	18
JOURNAL OF MEDICAL VIROLOGY	17
SCIENCE OF THE TOTAL ENVIRONMENT	16
COMPUTATIONAL AND MATHEMATICAL METHODS IN MEDICINE	15
FRONTIERS IN CELLULAR AND INFECTION MICROBIOLOGY	15
JOURNAL OF THE ROYAL SOCIETY INTERFACE	15
COMPUTERS IN BIOLOGY AND MEDICINE	14
FRONTIERS IN PHYSICS	14
NATURE COMMUNICATIONS	14
VACCINES	14

Supplementary Table S2 Authors' local impact measured by six indicators

Author	h_index	g_index	m_index	TC	NP	PY_start
WANG Y	14	44	2	2830	44	2020
WANG L	13	34	1.857	1346	34	2020
CHEN Y	11	38	1.571	2088	38	2020
LI X	11	29	1.571	848	32	2020
LI Y	11	17	1.571	301	43	2020
LIU X	11	29	1.571	848	30	2020
WANG J	11	24	1.571	621	36	2020
WANG X	11	27	1.571	778	54	2020
ZHANG L	11	32	1.571	1585	32	2020
ZHANG Y	11	32	1.571	1025	41	2020
ZHANG J	10	25	1.429	671	29	2020
GUO Y	9	14	1.286	769	14	2020
WANG H	9	22	1.286	518	26	2020
WANG S	9	13	1.286	193	26	2020
CHEN H	8	21	1.143	464	24	2020
CHEN X	8	18	1.143	328	26	2020
JIN Z	8	15	1.143	530	15	2020
LI M	8	16	1.143	406	16	2020
LIU S	8	17	1.143	991	17	2020
LIU Y	8	30	1.143	1615	30	2020
YANG J	8	13	1.143	183	20	2020
YANG X	8	14	1.143	210	17	2020
ZHAO Y	8	16	1.333	278	19	2021
BALEANU D	7	9	1	323	9	2020
KHAN A	7	12	1.167	333	12	2021
