# Supplementary figures and images for: SARS-CoV-2 and reproductive system: a scientometric study
Source: Front Reprod Health. 2026 May 14;8:1844245. doi: 10.3389/frph.2026.1844245 (PMC13216177; doi:10.3389/frph.2026.1844245)

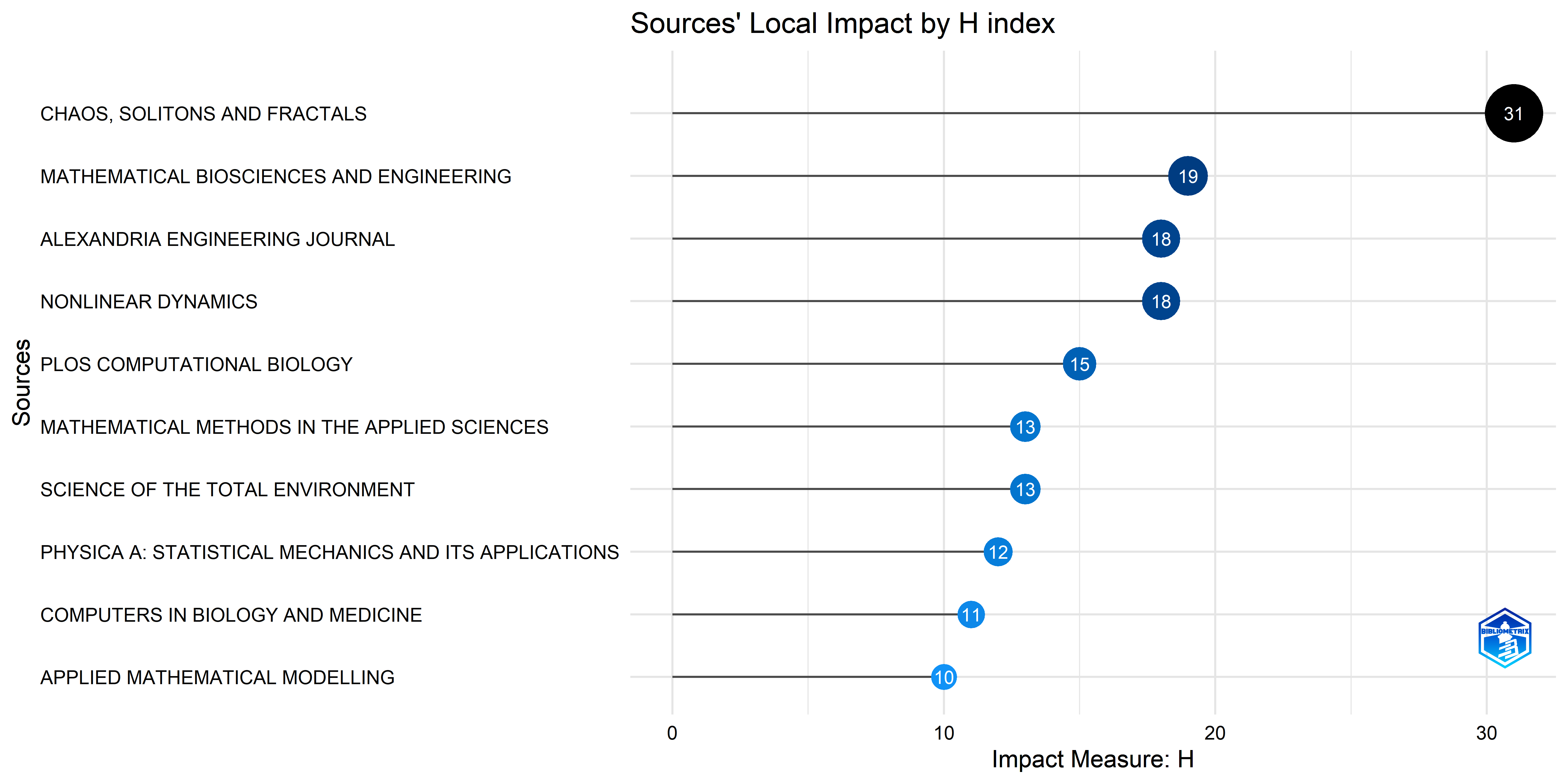

Supplement: Supplementary file 2 [file Image1.tif]

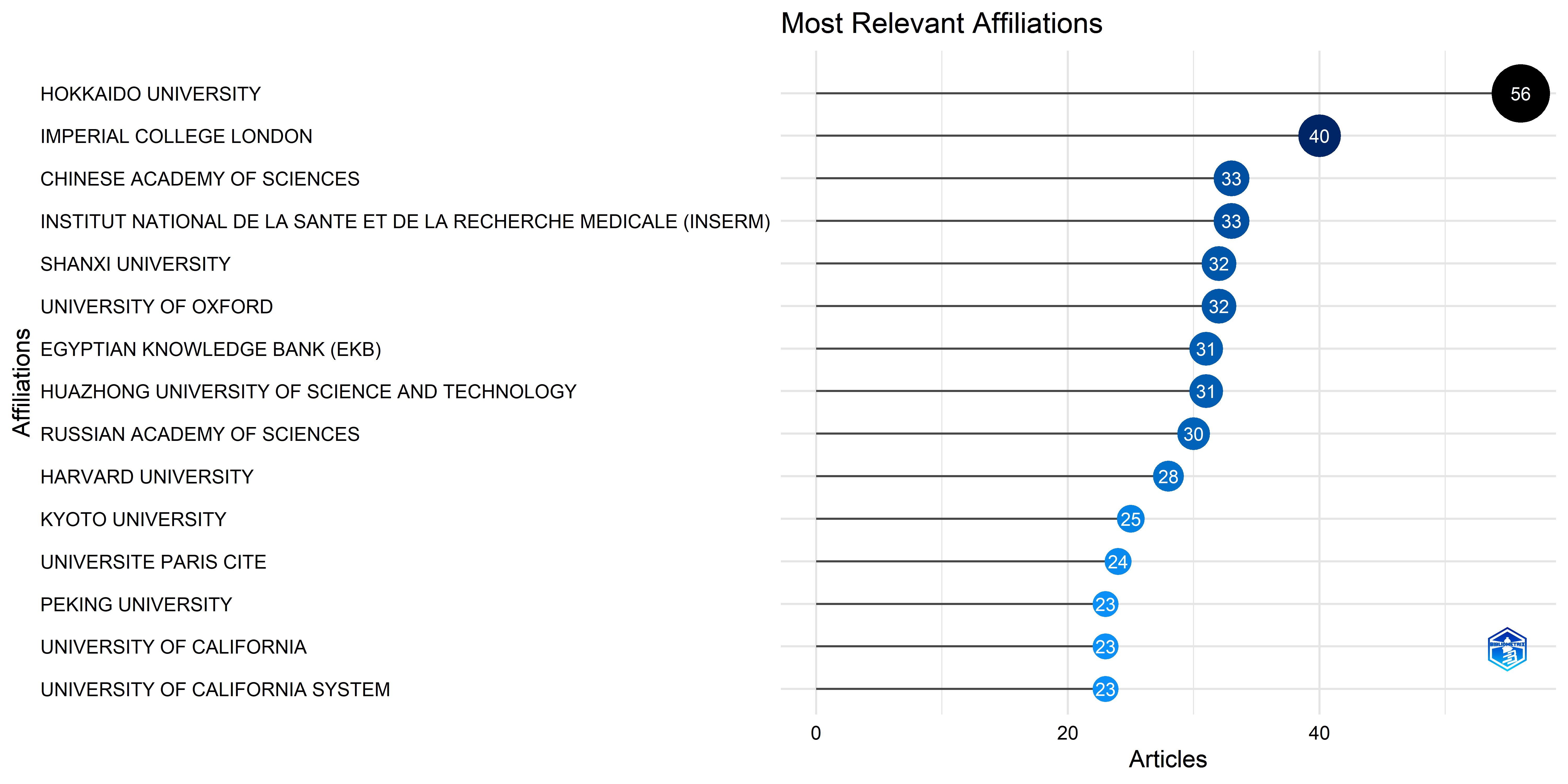

Supplement: Supplementary file 3 [file Image2.tif]

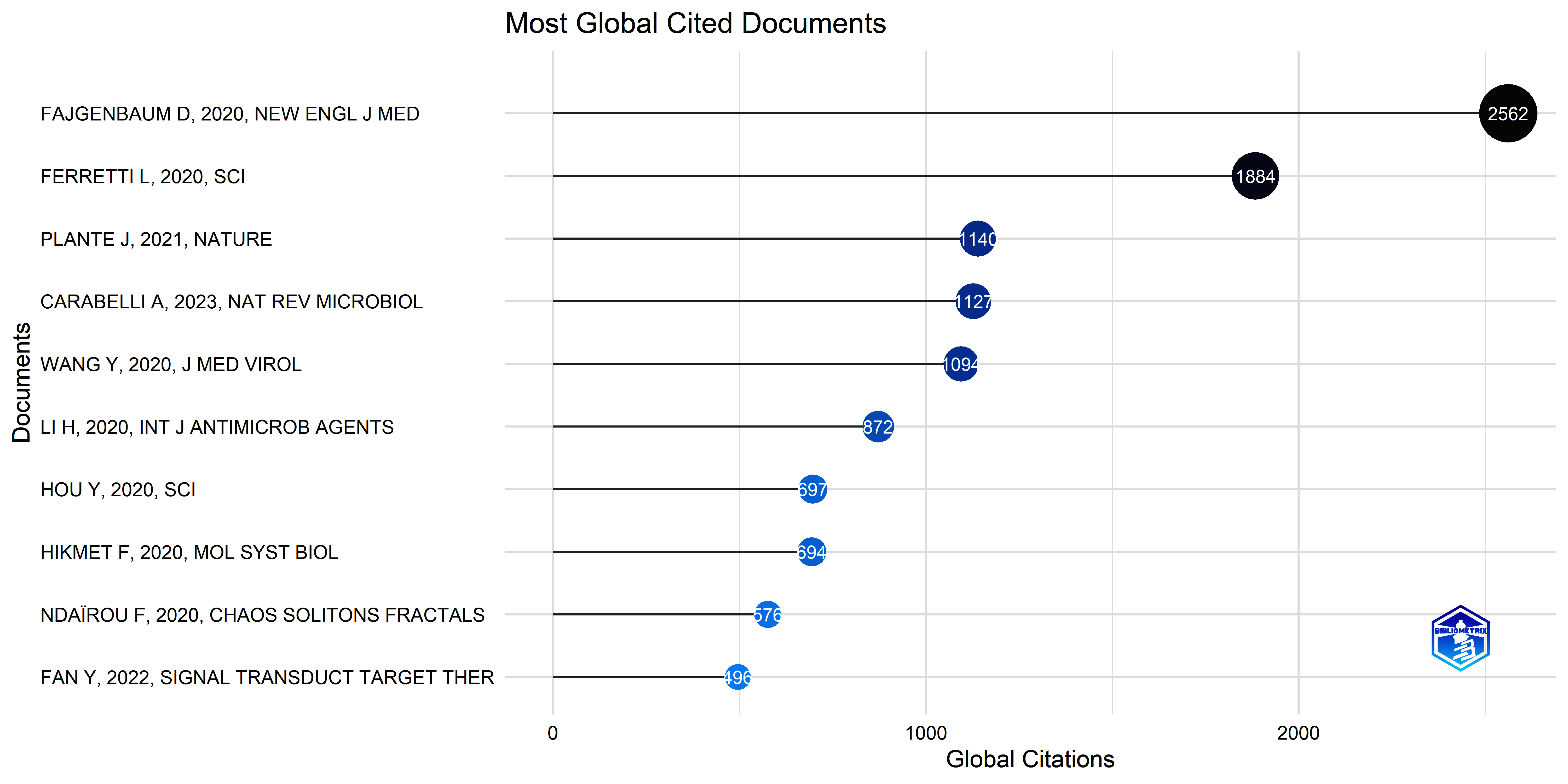

Supplement: Supplementary file 4 [file Image3.tif]

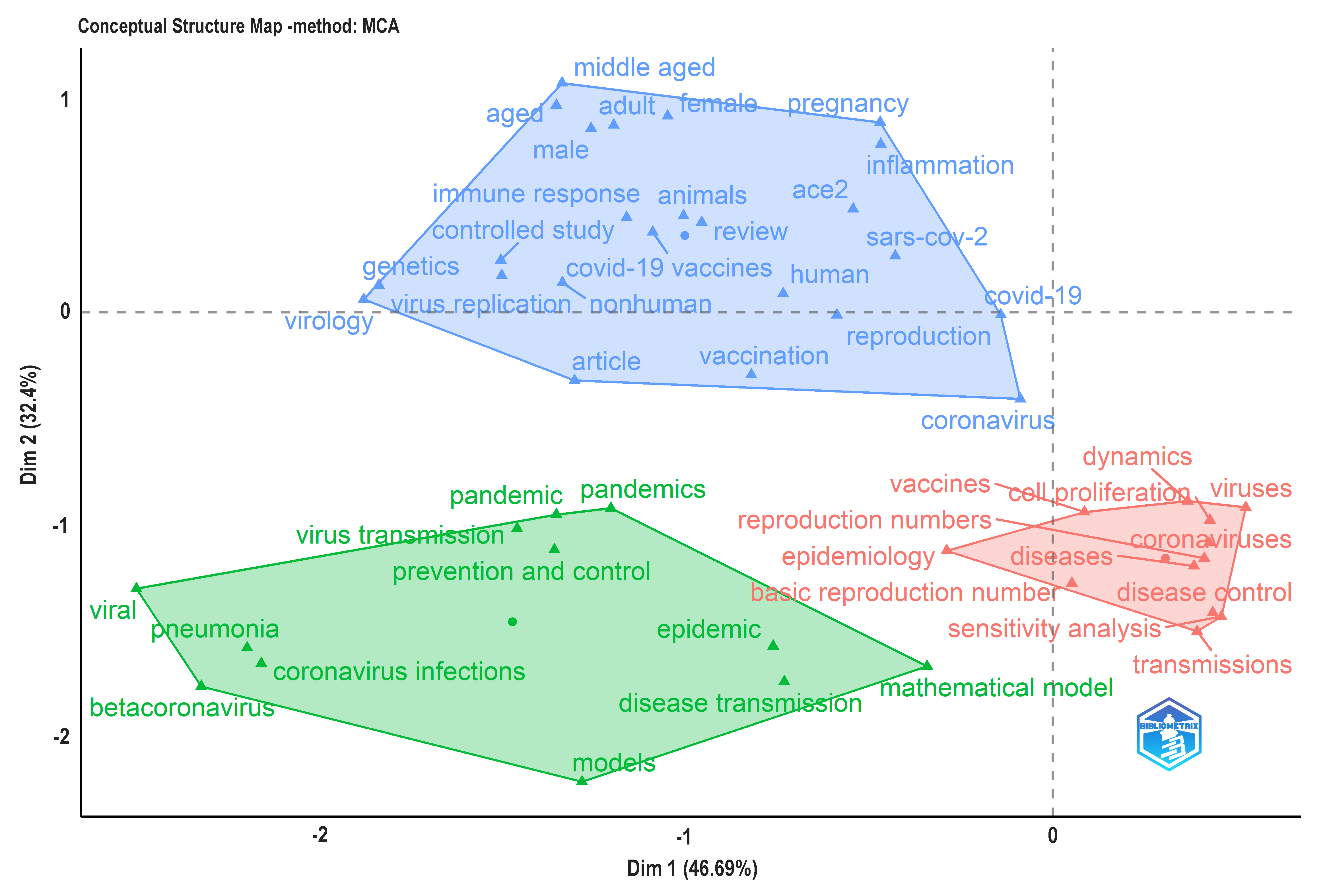

Supplement: Supplementary file 5 [file Image4.tif]

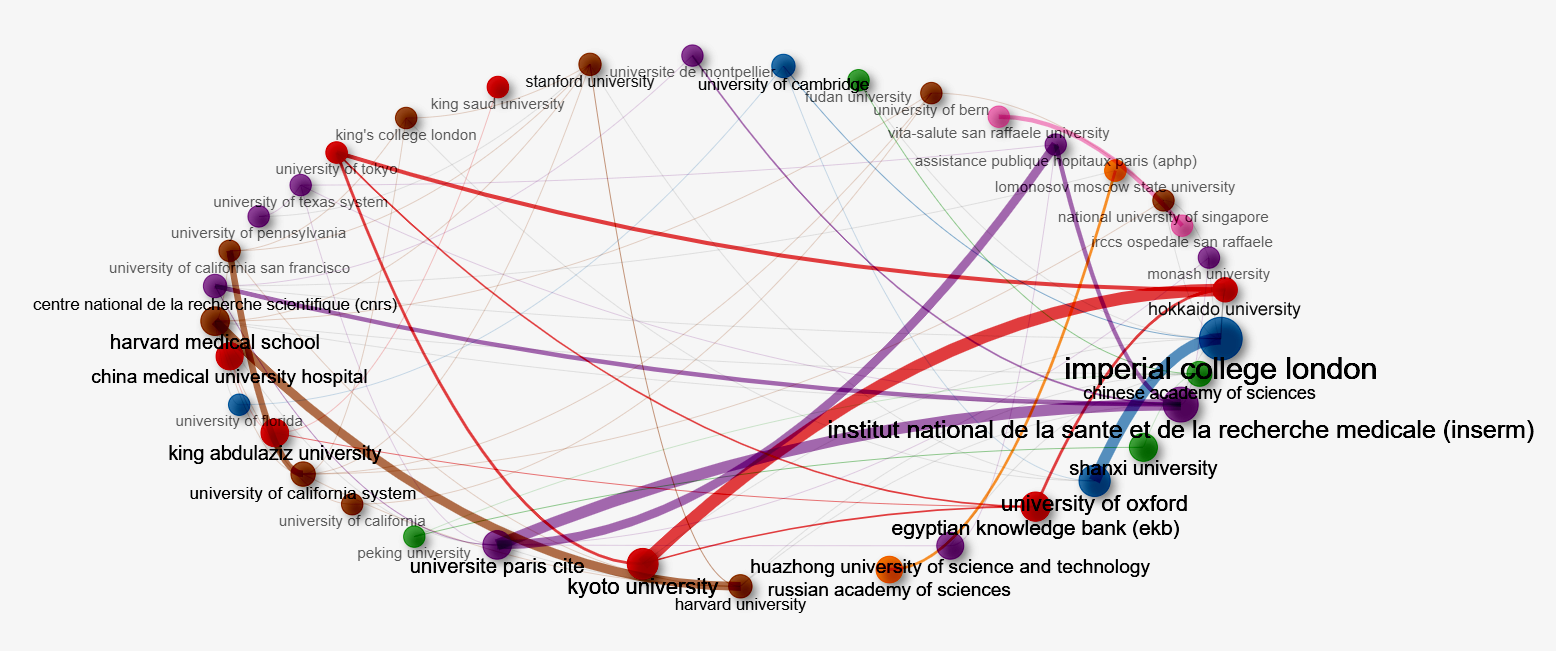

Supplement: Supplementary file 6 [file Image5.tif]

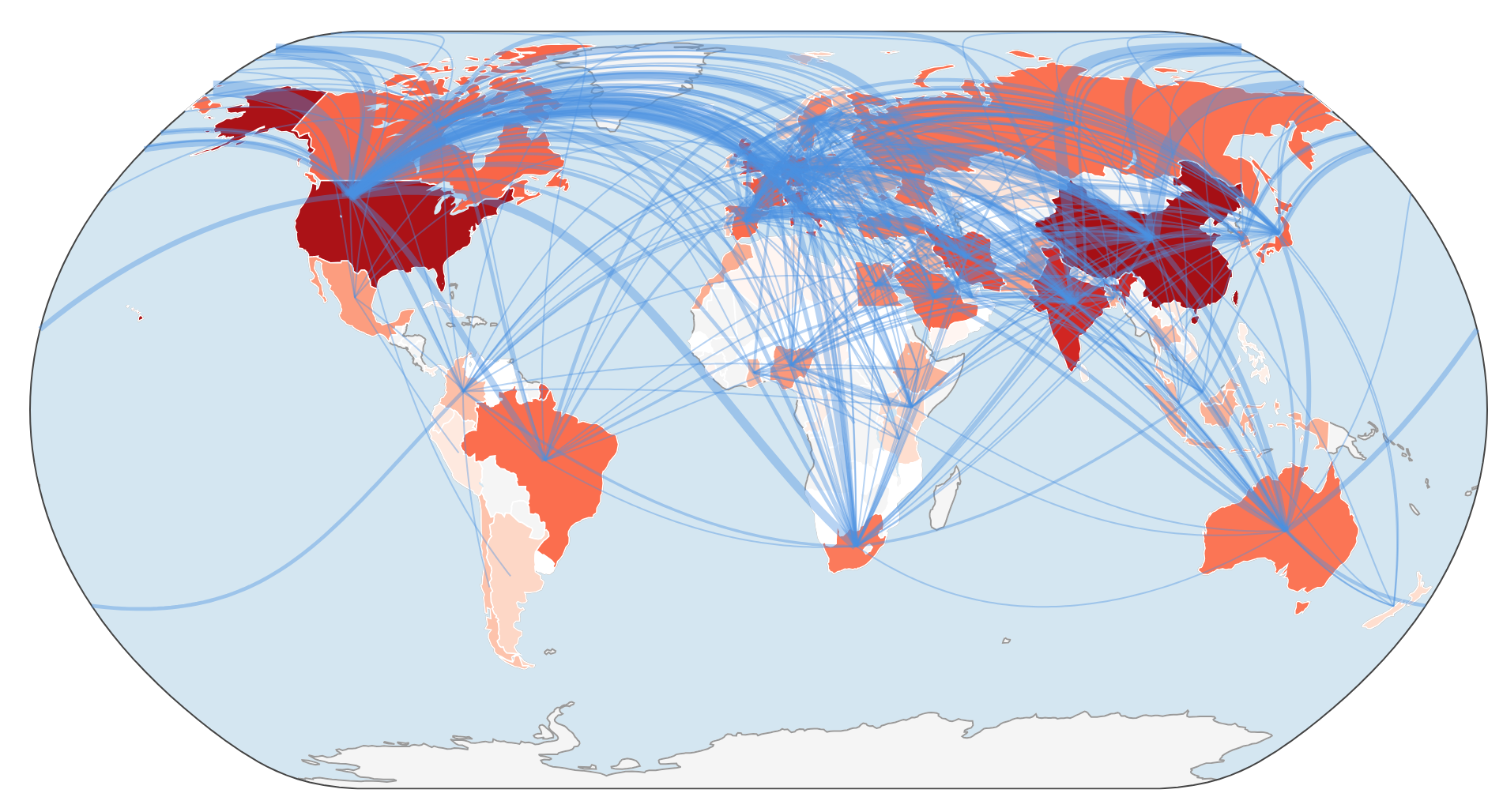

Supplement: Supplementary file 7 [file Image6.tif]
